# Identification and Validation of Reference Genes for qRT-PCR Studies of Gene Expression in* Dioscorea opposita*

**DOI:** 10.1155/2016/3089584

**Published:** 2016-05-26

**Authors:** Xiting Zhao, Xiaoli Zhang, Xiaobo Guo, Shujie Li, Linlin Han, Zhihui Song, Yunying Wang, Junhua Li, Mingjun Li

**Affiliations:** ^1^College of Life Sciences, Henan Normal University, Xinxiang 453007, China; ^2^Engineering Technology Research Center of Nursing and Utilization of Genuine Chinese Crude Drugs, University of Henan Province, Xinxiang 453007, China; ^3^Henan Province Engineering Laboratory of Green Medicinal Plant Biotechnology, Xinxiang 453007, China

## Abstract

Quantitative real-time polymerase chain reaction (qRT-PCR) is one of the most common methods for gene expression studies. Data normalization based on reference genes is essential for obtaining reliable results for qRT-PCR assays. This study evaluated potential reference genes of Chinese yam (*Dioscorea opposita* Thunb.), which is an important tuber crop and medicinal plant in East Asia. The expression of ten candidate reference genes across 20 samples from different organs and development stages was assessed. We identified the most stable genes for qRT-PCR studies using combined samples from different organs. Our results also suggest that different suitable reference genes or combinations of reference genes for normalization should be applied according to different organs and developmental stages. To validate the suitability of the reference genes, we evaluated the relative expression of* PE2.1* and* PE53*, which are two genes that may be associated with microtuber formation. Our results provide the foundation for reference gene(s) selection in* D. opposita* and will contribute toward more accurate gene analysis studies of the genus* Dioscorea*.

## 1. Introduction

Gene expression analysis has contributed to a better understanding of the function of candidate genes, which are involved in plant growth and development, as well as signal transduction and metabolism. Northern blotting, semiquantitative reverse transcription-PCR, and reverse transcription quantitative real-time PCR (qRT-PCR) [1] have each been used extensively in modern biological research. Among these, qRT-PCR has become a frequent first choice for gene expression studies, because of its high sensitivity, accuracy, and broad dynamic range [2–4]. Further, qRT-PCR has become the preferred method for many purposes such as clinical diagnosis, gene expression analysis in a specific tissue, or studies involving complex experiments and large numbers of genes [5–8]. Although it is widely used for gene expression analysis due to these advantages, qRT-PCR suffers from certain pitfalls such as differences in initial sample amount, RNA integrity issues, differences in the efficiency of cDNA synthesis, and differences in the overall transcriptional activity of the tissues or cells analyzed [3]; all of these factors can make the quantification of gene transcripts unreliable. To avoid bias, the selection of an appropriate normalization method becomes imperative for obtaining reliable quantitative gene expression results. The use of reference genes is commonly accepted as the most appropriate normalization strategy [9]. Consequently, the reliability of the quantitative results is highly dependent on the choice of appropriate reference genes for use in normalization.

Ideal reference genes should exhibit a constant level of expression in all tissues and development stages, independent of diverse experimental conditions. Moreover, reliable reference genes should have a transcript level similar to the target gene [10–13]. Housekeeping genes are commonly used as reference genes as they are typically supposed to have stable expression patterns. However, to date, more and more reports have demonstrated that there are no universally applicable reference genes with invariant expression levels, and inappropriate internal controls can lead to errors in the interpretation of biological data [14, 15]. Thus, there is an urgent need to systematically evaluate the stability of potential reference genes for particular experimental systems prior to adopting them for use in qRT-PCR normalization strategies. There have been a number of studies on the validation of reference genes in different plants including model plants (*Arabidopsis* [16, 17], rice [18, 19], tomato [20, 21], tobacco [22], etc.), crop plants (soybean [23, 24], pea [25], sugarcane [26], coffee [27, 28], peanut [29], cotton [2, 30],* Brassica napus* [15], wheat [31], etc.), vegetables (potato [8], chicory [32], cucumber [13], pepper [33], radish [34], etc.), fruits (berry [35], peach [36], banana [14], apple [37], etc.), flowers (petunia [38], rose [39, 40], etc.), tree plants, longan tree [41], and poplar [42]. Several algorithms such as geNorm [43], NormFinder [44], Best Keeper [45], and qBasePlus [46] have been developed to evaluate the most stable reference genes from among an input panel of candidate genes for a given set of experimental conditions.

Chinese yam (*Dioscorea opposita* Thunb.) is an important food and medicinal plant that is widely cultivated in East Asia. Modern scientific research has revealed that* D. opposita* has great health benefits [47–51]. Chinese yam is widely used as an ingredient in functional foods and drugs that enjoy great popularity in East Asia. Given its important medical and edible value, there is growing interest in this plant from the food and other industries. Thus, the production of* D. opposita* is gaining more attention. However, it is known that long-term vegetative propagation of* D. opposita* leads to serious virus infections and consequent losses in yield and quality. There is a desperate demand for improved resources and methods for high quality* D. opposita* production.

Propagation via virus-free plantlets can effectively solve the problems mentioned above, but the low survival rate and inconvenience of transporting these plantlets hinder the promotion and application of such methods. Propagation via microtubers and protocorm-like bodies (PLBs) that are induced from virus-free plantlets can overcome such shortcomings and is gaining attention rapidly. Microtubers are small tubers originating from tissues* in vitro*; they are more tolerant to stress conditions than plantlets and do not have the requirement of frequent subculturing as is needed for plantlet maintenance. Therefore, microtubers are attractive alternative to* in vitro*-grown plantlets for use as a means of micropropagation and exchange of healthy* D. opposita* materials. PLBs are undifferentiated tissue masses that outwardly resemble somatic embryos in form and development [52]; they have great potential for use as target explants for high frequency regeneration/propagation. The rooting of PLBs is endogenous and can resolve the problem of low survival rates that is known to be a problem with plantlets cultured* in vitro* [53]. The molecular mechanisms underlying the formation of microtubers and PLBs remain as yet unclear. Investigation of the key genes in microtuber and PLB formation will enable researchers to gain insight into the molecular basis of these processes. Some candidate genes related to microtuber formation in* D. opposita* were recently identified by transcriptome sequencing, and we have cloned genes involved in PLB formation in* D. opposita*. However, there has to date been little focus on which reference genes are well-suited for studies of microtuber and PLB formation in* D. opposita*; no well-defined and validated set(s) of reference genes have been described for* D. opposita*.

In this study, we evaluated ten different reference genes (*ACT*,* APT*,* EF1-α*,* GAPDH*,* TUB*,* UBQ*,* TIP41*,* MDH*,* PP2A*, and* GUSB*) for their potential for use as internal normalization controls in* D. opposita*. The expression patterns of these reference genes were tested in a range of organ types and developmental stages to identify the most suitable genes for use as internal controls in qRT-PCR expression studies in* D. opposita*.

## 2. Materials and Methods

### 2.1. Plant Materials and Treatments

Experiments were performed using* D. opposita* cv. Tiegun. Germ-free materials were maintained as described previously [54]. PLBs were produced from nodal segments in Murashige and Skoog (MS) basal medium [55] supplemented with 3 mg L^−1^ thidiazuron, 3 mg L^−1^ kinetin, and 30 g L^−1^ sucrose (our unpublished results); microtubers were produced as described previously [56].

Materials from three separate organs, including root, stem, and leaf, were collected at the same time (42 d after transfer to media); three different “sections” for each of the three organs were also collected: an upper part, a middle part, and a lower part. For PLBs, samples were collected at four different stages (our unpublished data). The first stage is the explant stage, during which a nodal segment is inoculated and the culture is initiated, named PLBs-0; the second stage is the explant swelling stage; the white small protrusions appear at leaf axils during this stage, named PLBs-I; the third stage is the primary PLB stage; white small protrusions become large and turn into light green PLBs cluster during this stage, named PLBs-II; the 4th stage is the mature PLB stage; the light green PLBs cluster grows up and becomes ivory coloured, named PLBs-III. For PLBs, samples were collected at four different stages. For microtubers, samples were collected at seven different stages as previously described by Li et al. [56]: initial explant stage (DMT-0), budding stage (DMT-1), bud stretching stage (DMT-2), bud visible swelling stage (DMT-3), microtuber initiation stage (DMT-4), microtuber enlargement stage (DMT-5), and microtuber mature stage (DMT-6). All samples were frozen in liquid nitrogen immediately after harvesting and stored at −80°C until future use.

### 2.2. RNA Extraction and cDNA Synthesis

Total RNA was extracted from different samples with a TaKaRa Mini-BEST Plant RNA Extraction Kit (TaKaRa, Japan), including DNase I treatment, according to the manufacturer's instructions. The integrity of the total RNA was checked on 1% agarose gels and RNA quantity and purity were evaluated by measuring the optical density at 260 nm and the A260/280 absorption ratio using a NanoDrop 2000 spectrophotometer (Thermo Fisher Scientific, US). cDNA synthesis was performed using HiScript*™* Q Select RT SuperMix for qPCR (Vazyme, China) following the manufacturer's instructions. For all samples, about 400 ng of total RNA was used to generate cDNA samples using Oligo(dT)_18_ primer in a final reaction volume of 10 *μ*L. The cDNA products were diluted 10-fold with nuclease-free water prior to use in the qRT-PCR assays.

### 2.3. Selection of Candidate Reference Genes

Based on previous reports of the effective application of reference genes for use in studies of various plant species [8, 16–18, 23, 35, 36, 41, 42], we selected ten candidate reference genes for an investigation to identify the most stably expressed genes in various organs and developmental stages (Table 1). The* ACT* and* GAPDH* gene sequences were obtained by degenerate PCR and have been submitted to the National Center for Biotechnology Information (NCBI, USA), with accession numbers of KR361321 and KR361320, respectively (Supplemental Document  1, in Supplementary Material available online at http://dx.doi.org/10.1155/2016/3089584). The sequences of the eight other candidate reference genes, including* TUB*,* TIP41*,* UBQ*,* PP2A*,* MDH*,* GUSB*,* APT*, and* EF1-α*, were selected from our transcriptome database that was generated via high-throughput Illumina sequencing (Novogene, China, Supplemental Document  1). These ten genes belong to different functional classes and were thus chosen to reduce the chance of the occurrence of coregulated expression among the candidates.

### 2.4. Design and Validation of qRT-PCR Primers

Specific primers were designed for qRT-PCR analysis using Primer 5 software (the sequences are listed in Table 1) with melting temperatures (*T*_m_) of 55–61°C, primer lengths of 17–25 bp, 40–60% GC content, and amplicon lengths of 80–200 bp. An exception to this was the amplicon length for the* MDH* primers, which was 224 bp.

qRT-PCR was carried out in 96-well plates with an ABI 7500 Real-Time PCR System and 7500 System Software (Applied Biosystems, Alameda, CA, USA) using SYBR Green-based PCR assays. Each reaction mix contained 1 *μ*L of 10-fold diluted cDNA, 10 *μ*L of EvaGreen 2x qPCR MasterMix (Applied Biological Materials Inc., Canada), 0.6 *μ*L of each primer, and nuclease-free water to a final volume of 20 *μ*L. The thermal cycling program was as follows: 95°C for 10 min, followed by 40 cycles of 95°C for 15 s and 60°C for 60 s in 96-well optical reaction plates (Bio-Rad, USA). The melting curves were analyzed at 60–95°C after 40 cycles. The amplification products were checked on 3% agarose gels. Each qRT-PCR analysis was performed with three technical replicates.

The qRT-PCR efficiency was determined for the ten candidate reference genes and the two target genes based on the slope of a linear regression model [57]. For this, cDNA from different samples was bulked and then used as the PCR template. The corresponding qRT-PCR efficiencies (*E*) were calculated according to the equation

(1) $$\begin{array}{c} E=\left[\;10^{\left(1/-\text{slope}\right)}-1\;\right]\times 100\% \end{array}$$

(see [58]).

For each gene, PCR efficiency was determined by measuring the mean threshold cycle (Ct) to a specific threshold [59, 60] for serial dilution of bulked cDNA. Five-point standard curves of a tenfold dilution series (1 : 1, 1 : 10, 1 : 100, 1 : 1000, and 1 : 10000) from pooled cDNA were used to calculate PCR efficiency. A standard curve was repeated in three dependent plates. The primer sequences and amplicon characteristics including *T*_m_, length, amplification efficiency with standard deviation (SD), and correlation coefficients of the ten candidate reference genes are listed in Table 1.

### 2.5. Statistical Analysis

The stability of the expression of each candidate gene was analyzed using the geNorm version 3.5 [43] and the NormFinder version 0.953 [44] using settings suggested in the geNorm and NormFinder user manuals. The Ct values were converted into relative expression values for the genes, and these values were used as the input for the geNorm and NormFinder programs. All other statistical analyses were performed with Microsoft Excel 2010.

### 2.6. Normalization of* PE2.1* and* PE53*

To test whether the choice of different reference genes influenced the final normalized results, the expression patterns of* PE2.1* (comp86916_c0) and* PE53* (comp76893_c0) were analyzed in parallel.* PE2.1* and* PE53* are members of the pectinesterase (PE) superfamily. According to our transcriptome analyses of microtuber induction and formation of* D. opposita* (unpublished findings), these are likely to be genes associated with the regulation of the starch and sucrose metabolism and signaling pathways. Therefore, they may play important roles in microtuber formation. The primer pairs used for the qRT-PCR analysis of these genes are listed in Table 1. The relative expression levels of* PE2.1* and* PE53* were estimated at three different development stages (DMT-0, DMT-3, and DMT-4) of microtuber formation, using five different normalization approaches: (1) with the most stable reference gene (*APT*); (2) with the two most stable reference genes selected by geNorm (*TUB* and* UBQ*); (3) with the two most stable reference genes identified by NormFinder (*ACT* and* APT*); (4) with the four reference genes suggested by both analyses (*TUB*,* UBQ*,* ACT*, and* APT*); (5) with picking up the least stable gene to act as a reference (*GUSB*). The relative expression of the target gene was calculated using the 2^−ΔΔCt^ method [61]. The mRNA levels of the target genes at DMT-0 were employed as a calibrator and were set to 1.

## 3. Results

### 3.1. RNA Quality Check

The quality of RNA samples is critical for successful gene expression analysis. We extracted total RNA from 20 different plant materials. Only RNA samples with both 28S and 18S ribosomal RNA bands with a density ratio of about 2.0 and without smears on 1% agarose gels were used for subsequent analysis. The value of the A260/280 ratio for all of the RNA samples was between 2.0 and 2.2 and the A260/230 ratio values for all samples were higher than 2.0, indicating that the RNA samples were of sufficient purity for use in qRT-PCR assays, without contamination.

### 3.2. Verification of Primer Specificity and PCR Efficiency Analysis

**Figure 1:**
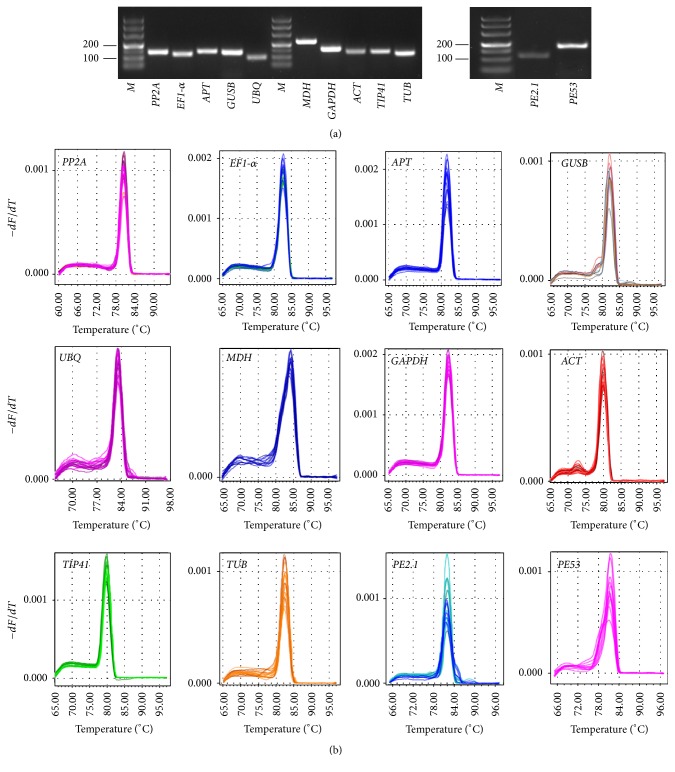
Specificity of qRT-PCR amplicons. (a) Agarose gel electrophoresis showing amplification of a single product of the expected size for each candidate gene and two target genes for reference gene validation. *M* represents DL2,000 DNA marker. (b) Dissociation curves with single peaks generated for all amplicons.

To check the specificity of the primers for these candidate reference genes and two target genes, agarose gel electrophoresis and melting curve analyses were performed following completion of the qRT-PCR assays. The results of the agarose gel electrophoresis indicated that all of the primer pairs amplified a single band of the expected respective size and formed no primer dimers or other nonspecific amplification products (Figure 1(a)). The specificity of the amplicons was further confirmed by the observation of a single peak in the melting curve analyses after 40 cycles (Figure 1(b)). The PCR amplification efficiency for the ten reference and the two target genes varied from 95.536% for* ACT* to 103.415% for* TIP41*, and correlation coefficient (*R* ^2^) values ranged from 0.966 to 0.998 over 10^4^ times of cDNA dilution for* PE2.1* and* ACT*, respectively (Table 1).

### 3.3. Expression Profile of the Candidate Reference Genes

**Figure 2:**
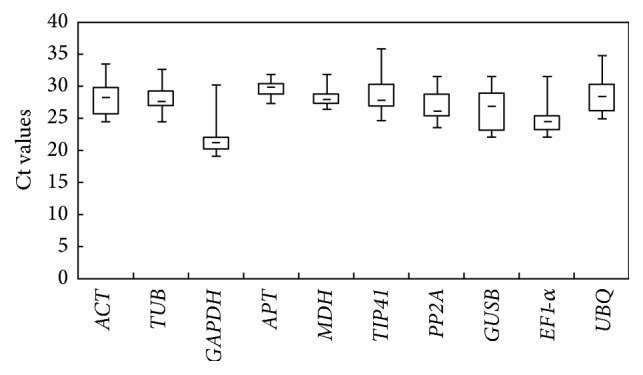
RNA transcription levels of reference genes tested, presented as Ct mean value in the different samples. Boxes indicate the 25th/75th percentiles, lines across the boxes depict the medians, squares represent the means, and whiskers indicate the ranges for all samples.

Ct values were used to analyze the steady-state mRNA levels of each reference gene [60]. To obtain reliable results, all qRT-PCR assays were conducted with three technical replicates, and the mean values were used for expression profile analysis. Analysis of the Ct values across all samples indicated differences in the expression among the candidate reference genes (Figure 2), suggesting varying levels of transcript abundance for the ten genes analyzed. In all tested samples, the lowest mean Ct value (22.30) was exhibited by* GAPDH*, indicating the highest abundance among the reference genes, whereas* APT* showed lower levels of expression (mean Ct = 29.65).* GAPDH* and* EF1-α* showed higher expression levels than other genes in all tested samples, with the average Ct values ranging from 22.30 to 24.83, while the other genes had lower expression levels, with average Ct values ranging from 26.36 to 29.65. The smallest variation in gene expression was observed for* APT* (4.47), while* GAPDH* (11.07) and* TIP41* (10.88) were the genes with the most variable levels of expression. These results indicated that none of the selected genes had a constant level of expression in the different* D. opposita* samples tested. Therefore, it is extremely important to evaluate the suitability of particular reference genes for use in gene expression normalization under particular experimental conditions.

### 3.4. Evaluation of the Expression Stability of Candidate Reference Genes

Since the 10 candidate reference genes showed wide variations in expression levels in the different samples, it was necessary to use statistical methods to rank the stability of expression of the 10 genes and determine the number of reference genes necessary for accurate gene expression profiling under a given experimental condition. Two different analysis programs, geNorm and NormFinder, were used in the following analysis. The raw Ct values were manually transformed into the geNorm and NormFinder data input formats and then analyzed by geNorm and NormFinder.

**Figure 3:**
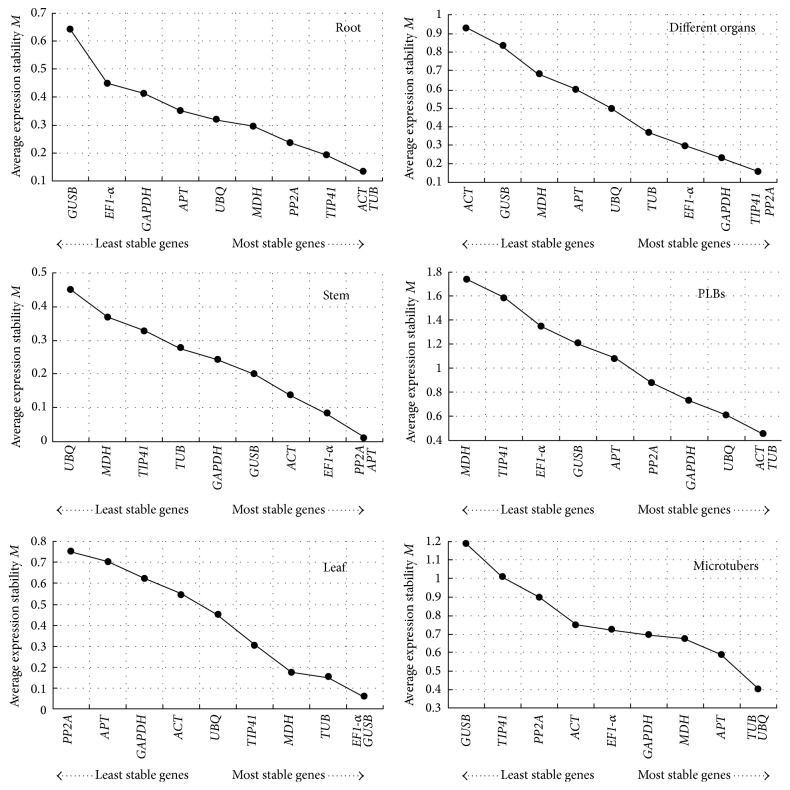
Gene expression stability and rankings of ten candidate reference genes, as calculated by geNorm software. The average expression stability (*M*) was calculated following stepwise exclusion of the least stable gene across all the samples within an experimental set. The lowest *M* value indicates the most stable gene, while the highest value represents the most highly variable gene.

In the geNorm program, the average expression stability (*M*) value for each gene was calculated based on the average pairwise variation between all genes tested. Stepwise exclusion of the least stable gene allows the genes to be ranked according to their *M* value (the lower the *M* value, the higher the gene's expression stability). The results obtained with the geNorm program are presented in Figure 3. Among the ten candidate reference genes, none of the reference genes had identical expression under different experiment conditions. The* ACT* and* TUB* genes ranked the highest for the different development stages of PLB formation, with an *M* value of 0.452. The* TUB* and* UBQ* genes proved to be the best candidates for normalization in samples at different development stages during microtuber formation, with an *M* value of 0.399. For root samples, the most stable genes were* ACT* and* TUB,* with an *M* value of 0.131. For stems,* PP2A* and* APT* were the most stably expressed, with an *M* value of 0.01. For leaves,* EF1-α* and* GUSB* exhibited the most stable expression, with an *M* value of 0.059. When different organ sample sets (root, stem, or leaf) were analyzed together,* TIP41* and* PP2A* were found to be the most stably expressed genes, with an *M* value of 0.155.

**Figure 4:**
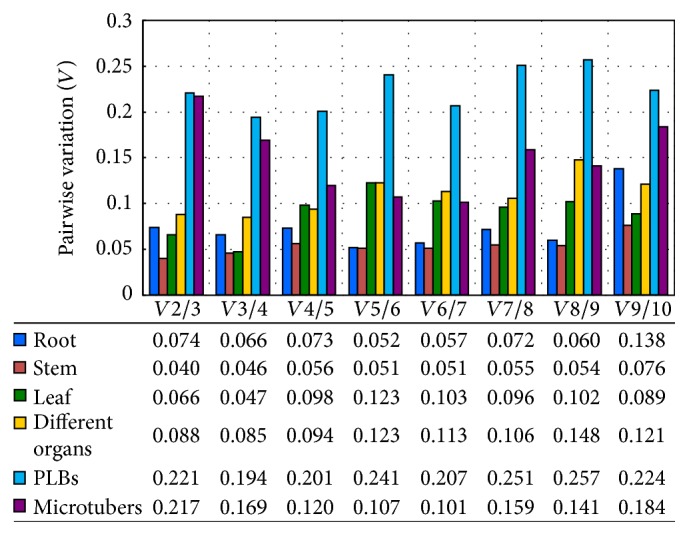
Pairwise variation (*V*) analysis of the candidate reference genes. The pairwise variation (*Vn*/*n* + 1) was analyzed between two sequential normalization factors NF*n* and NF*n* + 1 that contained an increasing number of reference genes using geNorm software. *Vn*/*n* + 1 < 0.15 indicates that the inclusion of an additional reference gene is not required.

The geNorm program was also used here to calculate the optimal number of reference genes required for accurate normalization in the different sample sets. The software determines the pairwise variation *Vn*/*n* + 1, which measures the effect of adding further reference genes on the normalization factor (which is calculated as the geometric mean of the expression values of the selected reference genes). It is advisable to add additional reference genes to the normalization factor until the added gene has no significant effect. A cut-off value of 0.15 has been widely accepted as the criterion for selecting a suitable number of reference genes, below which the inclusion of additional reference genes is not needed [43]. However, 0.15 is not an absolute cut-off value, but rather an ideal value. In our study, the *V*2/3 values of root, stem, leaf, and different organs were less than 0.15, suggesting that the optimal number of reference genes for normalization in these groups was at least two. For microtubers, the pairwise variation value of *V*3/4 was 0.169 and that of *V*4/5 was 0.120, suggesting that four reference genes were required (Figure 4).

NormFinder takes into account the intra- and intergroup variations for normalization factor (NF) calculations. The results for our study obtained with the NormFinder program are presented in Table 2. For the different development stages during PLB formation, the best combination recommended by NormFinder was* ACT* and* TUB*, and the most stable gene was* ACT*. Combination of* ACT* and* APT* was found to be the best during the different development stages of microtuber formation, in which the* APT* was the optimal reference gene. For the combined analysis of root, stem, and leaf, the best gene combination was* TIP41* and* EF1-α*, and the most stable gene* was TIP41*. The combination of* MDH* and* UBQ* was considered to be the best for leaves; if only one reference gene was to be used,* UBQ* would be the best choice for studies of leaves. The combination of* APT* and* GUSB* was found to be the most suitable for stems, and* GUSB* was the single most stable gene. For roots, the combination of* PP2A* and* TIP41* was the best combination, and* TIP41* was the most stable. In these cases, the most stable gene was consistent with the best combinations. No matter which of the programs was used, the most variable gene in roots was* GUSB*, the most variable gene in stems was* UBQ*, and the most variable gene in leaves was* PP2A*.* MDH* was the least stable gene during PLB formation.* GUSB* was the most variable in both the different organ samples and microtuber formation.

### 3.5. Reference Gene Validation in Gene Expression Study

**Figure 5:**
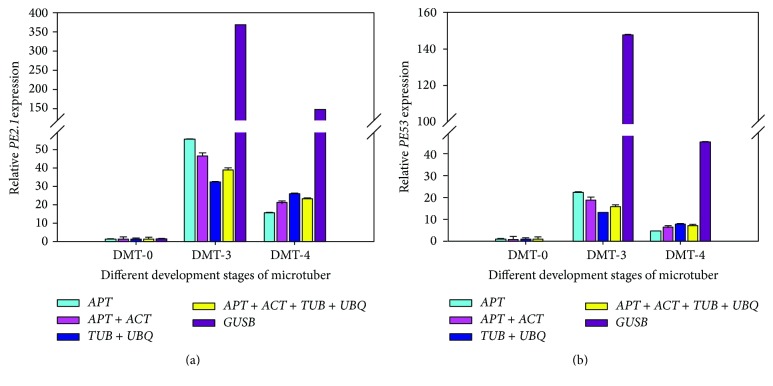
Relative expression levels of* PE2.1* (a) and* PE53* (b) during three different development stages of microtuber formation, normalized by different combinations of reference genes, as indicated.* APT* was found to be one of the most stable genes by both geNorm and NormFinder. The combination of* TUB* and* UBQ* was the optimal combination as selected by geNorm. The combination of* APT* and* ACT* was found to be optimal in the NormFinder analysis. The four most stable reference genes were suggested by both analyses. Standard error bars are indicated.

The expression of* D. opposita* genes encoding two PE enzymes was analyzed by qRT-PCR in order to validate the performance of the selected candidate genes as internal controls for normalization.* GUSB* was the most unstable gene for normalization according to the geNorm and NormFinder analysis. When* GUSB* alone was used as the reference, the expression level of* PE2.1* increased 368-fold at the bud visible swelling stage (DMT-3) as compared to its expression at the explants stage (DMT-0).* PE2.1* expression was increased 147-fold at the microtuber initiation stage (DMT-4) as compared to its expression at the explants stage (DMT-0).* APT* was considered to be one of the most stable genes by both the geNorm and the NormFinder programs. When* APT* was used as the reference gene, the expression levels of* PE2.1* were elevated 56- and 15-fold at the DMT-3 and DMT-4 stages, respectively, in comparison with the DMT-0 stage (Figure 5). Thus, the target gene expression profiles of the tested samples varied widely according to the reference gene chosen for normalization.

When the combinations of reference genes were used for normalization, a much more reliable expression profile for* PE2.1* was obtained (Figure 5(a)).* TUB* and* UBQ* were found to be the best combination by geNorm, and* PE2.1* expression in relation to this combination was consistent with that obtained with the employment of the two best reference genes indicated by NormFinder. Interestingly, the* PE2.1* expression profile as normalized by the best gene pairs was equivalent to those obtained when the four best reference genes identified by both programs were used. Similar results were observed for* PE53* (Figure 5(b)), suggesting that the use of two genes would be sufficient to get accurate and reliable normalization. These results further emphasized the necessity of evaluating reference gene stability before qRT-PCR analysis to avoid quantification errors.

## 4. Discussion

qRT-PCR has become a routine technique for gene expression studies, and normalization of qRT-PCR data with an appropriate internal control gene is essential to obtain results with biological relevance. Internal control genes must be selected with caution. However, it must be noted that there are no universal reference genes that are stably expressed under all biological materials and/or experimental conditions. Accordingly, the stability of reference genes needs to be verified prior to conducting qRT-PCR expression studies in* D. opposita*. In this study, we analyzed a large group of reference genes; suitable internal controls were identified for use in qRT-PCR analysis of different experimental conditions.

geNorm and NormFinder are two commonly used analysis programs for comparing the expression patterns of candidate reference genes and identifying the best suitable reference gene sets under particular conditions. Differences were observed in geNorm and NormFinder results for the best combinations of reference genes for each of the experimental conditions tested in this study (Table 2, Figure 3). This inconsistency between the two programs was expected, given that they are based on distinct algorithms. geNorm selects two genes with a low intragroup variation and approximately the same nonvanishing intergroup variation. In contrast, NormFinder selects the two best genes with minimal combined inter- and intragroup expression variation [44]. Both methods provide a stability value for each gene and select the best reference gene for normalization. In this study, only a few relevant differences were observed between the two methods. In addition, no matter how the ranking order for the separate results differed, the most unstable gene identified by both geNorm and NormFinder was the same in all sample sets, a finding that has been observed in other studies [2, 13, 20, 41]. Although the best combinations obtained from geNorm and NormFinder can obviously both be used as reference genes in experimental studies, we prefer the NormFinder results because geNorm is known to be influenced by the coregulation of reference genes [43].

In order to determine the suitable reference genes for obtaining accurate and reliable results in gene expression studies, we performed five different normalization approaches for target gene normalization. Significant differences were produced when the least stable gene was used for normalization. When the most reliable reference gene alone was used for normalization, the target gene expression pattern was in accordance with the best combinations of reference genes although there were still slight differences in expression profiles.

Vandesompele et al. [43] outlined, for the first time, a systematic survey of the errors related to the common practice of using only one reference gene. In the following years, increasing evidence has suggested that the application of more than one internal control gene should lead to more reliable results in gene expression studies [2, 62]. Although increasing the number of reference genes for normalization will improve the accuracy of the analysis, this is expensive and time-consuming. Therefore, the number of internal controls should be taken into account if the amount of RNA is limited or if a large number of samples need to be analyzed [41]. It has been suggested that the number of reference genes that need to be used is dependent on the considerations of a researcher's purpose [63]. The expression pattern analysis of* PE2.1* and* PE53* during microtuber formation emphasized the importance of the choice of the correct reference genes or gene combinations to achieve accurate qRT-PCR results (Figure 5). When single genes were individually used as reference genes, a large fluctuation in the results was observed. More reliable expression profiles of target genes could be obtained when using combinations of reference genes for normalization. According to our results, using two reference genes is a balance between accuracy, cost, and convenience. In addition, our results suggested that different suitable reference genes should be applied according to the different experimental conditions.

To the best of our knowledge, this is the first systematic validation of a set of candidate reference genes in* D. opposita* for the normalization of gene expression analysis using qRT-PCR. Our results provide the foundation for more accurate use of qRT-PCR in the analysis of gene expression in* D*.* opposita*. Further, our study will also benefit future gene expression studies in other species of the genus* Dioscorea*.

## 5. Conclusion

Our results suggest that different suitable reference genes or combinations of reference genes for normalization should be applied according to different experimental conditions.* EF1-α* and* TIP41* were identified as the most stable genes for combined studies of leaves, stem, and root, while* MDH* and* UBQ* were the best for leaves,* APT* and* GUSB* were the best for stems, and* PP2A* and* TIP41* were the best for roots. For PLB formation,* ACT* and* TUB* were the best reference genes. For microtuber formation,* ACT* and* APT* were considered to be the best combination.

## Supplementary Material

Supplemental Document 1 presents the sequences of the candidate reference genes.

## Figures and Tables

**Table 1:** Selected candidate reference genes and target genes for reference gene validation, primers, and amplicon characteristics.

Gene symbol	Gene name	Accession number/RNA-Seq number	Primer sequence (5′-3′)	Amplicon length (bp)	*T*_m_ (°C)	Amplification efficiency (%)	*R* ^2^
*TUB*	Beta-tubulin	comp80557_c0	CTTCGTGTTTGGGCAGTCT	116	60	101.205 ± 0.019	0.979
			CACAGTTCTCGGCCTCCTT				

*GAPDH*	Glyceraldehyde-3-phosphate dehydrogenase	KR361321	CAGCTCATTTGAAGGGTGG	156	60	98.269 ± 0.013	0.996
			CAAGAGGAGCAAGGCAGTT				

*ACT*	Actin	KR361320	CTCATTGATCGGCATGGAAGC	130	60	95.536 ± 0.007	0.998
			GGGGAACATAGTTGAACCACCAC				

*APT*	Adenine phosphoribosyltransferase	comp78359_c0	CAGGCAAAACAATTTCAGAAGC	137	60	99.376 ± 0.011	0.983
			AGCACGAAGTGTCCCACCA				

*MDH*	Malate dehydrogenase	comp84254_c0	GCCTTCCACAACCTTCAC	224	60	96.463 ± 0.012	0.994
			AGAATGGCAGCTCGTTCA				

*PP2A*	Protein phosphatase 2A subunit A3	comp74046_c0	ATGCCAATGTCTGGAAGTA	139	60	97.296 ± 0.007	0.986
			CGGTCAAGAGCACGAAT				

*TIP41*	TIP41-like family protein	comp79289_c0	TGTGCCAAATTCACCAA	133	60	103.415 ± 0.021	0.993
			CAAAACCACCTCATCATAGA				

*GUSB*	Beta-glucuronidase	comp87528_c0	GCCGAGCGGATGTAAGA	141	60	98.396 ± 0.016	0.973
			TGTTGTGAGTTGCCCTGT				

*EF1-α*	Elongation factor 1-alpha	comp77681_c0	ACTGTTCCTGTTGGTCGTG	122	60	99.426 ± 0.008	0.984
			TCTGGGAGGGATTCGTG				

*UBQ *	Ubiquitin	comp74534_c0	GGGCTTTCAAGGTCGTC	100	60	99.378 ± 0.009	0.971
			TGAAGGGTTTGCTCATCC				

*PE2.1*	Pectinesterase 2.1	comp86916_c0	GATCAACAGGTGCAACATCGAG	116	60	98.395 ± 0.019	0.966
			CGGCGTCACCAAAGATGAAG				

*PE53*	Pectinesterase 53	comp76893_c0	CTAAGGGATGGTATAACTGGGGAG	175	60	99.397 ± 0.016	0.991
			CAATCAGAGCCGTCAATGAAGC				

*R* ^2^: correlation coefficient.

**Table 2:** Candidate reference genes ranked according to their expression stability as determined by NormFinder.

Root		Stem		Leaf		Different organs		PLBs		Microtubers	
Ranking	Stability value	Ranking	Stability value	Ranking	Stability value	Ranking	Stability value	Ranking	Stability value	Ranking	Stability value
*GUSB*	0.011	*UBQ*	0.005	*PP2A*	0.007	*GUSB*	0.009	*MDH*	0.012	*GUSB*	0.008
*EF1-α*	0.004	*MDH*	0.004	*GAPDH*	0.006	*ACT*	0.008	*EF1-α*	0.011	*GAPDH*	0.006
*GAPDH*	0.004	*TIP41*	0.003	*APT*	0.005	*APT*	0.006	*TIP41*	0.009	*TIP41*	0.004
*APT*	0.003	*GAPDH*	0.003	*TIP41*	0.005	*UBQ*	0.006	*PP2A*	0.008	*PP2A*	0.004
*UBQ*	0.003	*TUB*	0.002	*GUSB*	0.004	*MDH*	0.005	*GUSB*	0.006	*TUB*	0.003
*MDH*	0.002	*ACT*	0.002	*ACT*	0.003	*GAPDH*	0.005	*GAPDH*	0.006	*UBQ*	0.003
*TUB*	0.002	*EF1-α*	0.002	*TUB*	0.003	*TUB*	0.001	*APT*	0.004	*ACT*	0.002
*ACT*	0.001	*PP2A*	0.001	*EF1-α*	0.003	*PP2A*	0.001	*UBQ*	0.002	*EF1-α*	0.002
*PP2A*	0.001	*APT*	0.001	*MDH*	0.003	*EF1-α*	0.001	*TUB*	0.002	*MDH*	0.002
*TIP41*	0.001	*GUSB*	0.001	*UBQ*	0.003	*TIP41*	0.001	*ACT*	0.001	*APT*	0.001

Best combination	Stability value	Best combination	Stability value	Best combination	Stability value	Best combination	Stability value	Best combination	Stability value	Best combination	Stability value

*PP2A/TIP41*	0.001	*APT/GUSB*	0.001	*MDH/UBQ*	0.002	*EF1-α/TIP41*	0.000	*ACT/TUB*	0.001	*ACT/APT*	0.001

Most stable	Stability value	Most stable	Stability value	Most stable	Stability value	Most stable	Stability value	Most stable	Stability value	Most stable	Stability value

*TIP41*	0.001	*GUSB*	0.001	*UBQ*	0.003	*TIP41*	0.001	*ACT*	0.001	*APT*	0.001

## Abbreviations

ACT: Actin
APT: Adenine phosphoribosyltransferase
EF1-*α*: Elongation factor 1-*α*
GAPDH: Glyceraldehyde-3-phosphate dehydrogenase
GUSB: Beta-glucuronidase
MDH: Malate dehydrogenase
MS: Murashige and Skoog (medium)
PE: Pectinesterase
PP2A: Phosphoprotein phosphatase
TIP41: TIP41-like family protein
TUB: Beta-tubulin
UBQ: Ubiquitin.
