# Supplementary material for: Identification and Validation of Reference Genes for qRT-PCR Studies of Gene Expression in Dioscorea opposita
Source: Biomed Res Int. 2016 May 26;2016:3089584. doi: 10.1155/2016/3089584 (PMC4899605; doi:10.1155/2016/3089584)
Supplement: Supplementary file 1 — Supplemental Document 1 presents the sequences of the candidate reference genes. [file 3089584.f1.doc]

**Supplemental Document 1**

**TUB**

**>comp80557_c0**

TTAATACACTATAATAAATATGTATAGAAACAGTGCAAACCAGTGCTTATTAGTTTCAAAATGATATGCATGACAATGAATGAGAACAACATATATAATTAGACTTGGTTCAAAAACTAGACTATACATTGATAGCGAAGACAAAACAAACAAATGCTTTCAGAAGCAACAAAGCTCAAAAACAGGAAGCCGATGGATAAAAAAACAGGTGGCACATTAAGTCAGACTCTAAAAGCAGAAACAACAGCACATCCAGGGAATGTCATACAAGCCATACACAAACATTAGGAGTACAAATATAATGATGTCTATCTCTCAAGATTAACAAGATGATATTAAGCTCCTTTCACAACCCCACCCAAAATACATGGAAAAACACCAGACAACACATATCAAGCTTGGACTTCCTCCTCTTCCTCCTCTTCGTACTCTTCATCGGCTGTTGCATCCTGGTACTGCTGGTACTCGGCAACAAGATCATTCATGTTGCTCTCAGCCTCAGTGAATTCCATTTCATCCATACCCTCACCAGTGTACCAATGCAAGAATGCCTTCCTCCTGAACATGGCGGTGAACTGCTCGCTAACCCTGCGGAACATCTCCTGGATGGAGGTTGAGTTGCCGATAAATGTGGATGCCATCTTGATGCCCTTTGGCGGTATGTCACACACACTGGACTTGACATTGTTGGGGATCCATTCCACAAAATAGGAAGAATTCTTGTTCTGAACATTGATCATCTGCTCATCAACTTCCTTGGTGCTCATCTTTCCACGGAACATGGCAGAGGCAGTGAGGTAGCGGCCATGTCGGGGGTCAGCAGCACACATCATGTTCTTGGAGTCCCACATCTGTTGGGTGAGCTCAGGTACAGTCAGGGCCCGATACTGCTGTGAGCCCCTCGAGGTGAGAGGTGCAAAACCAACCATGAAAAAGTGGAGACGTGGGAATGGGATCAAGTTCACGGCAAGCTTGCGAAGATCGGAGTTGAGCTGACCAGGGAACCGGAGGCAGCATGTGATGCCACTCATGGTGGCAGAGATCAGATGATTAAGATCGCCAAAAGTTGGAGTTGCAAGCTTGAGAGTCCGGAAGCAGATATCATAGAGAGCTTCATTGTCAAGGACCATGCACTCATCAGCATTCTCAACAAGTTGATGAACAGAAAGTGTAGCATTGTATGGCTCCACAACAGTGTCAGACACCTTGGGTGATGGGAAGACAGAGAAGGTGAGCATCATCCGGTCAGGGTACTCCTCCCTTATCTTAGAGATGAGAAGGGTGCCCATGCCAGATCCTGTGCCTCCTCCCAAAGAATGACATACTTGGAAACCTTGCAGGCAGTCGCAGTTCTCGGCCTCCTTGCGAACGACATCGAGCACAGAATCAATAAGCTCAGCACCCTCCGTGTAGTGCCCCTTCGCCCAGTTGTTGCCGGCACCGGACTGACCGAAGACGAAGTTATCAGGCCTAAAGATCTGGCCAAAAGGACCAGATCGAACGGAGTCCATGGTGCCTGGCTCAAGGTCCATGAGAACGGCGCGGGGAACGAAGCGACCGCCGCTGGCCTCGTTGTAATAGACATTGATGCGTTCAAGCTGGAGGTCAGAGTCGCCATTATACTTTCCGGTGTGATCGATGCCGTGCTCGTCGCAAATCACCTCCCAGAACTTGGCTCCGATCTGGTTGCCGCACTGCCCTCCCTGGATGTGCAGGATCTCTCTCATCGTCGATGATCGGAAGGTCGTGGACGCGCCGGCGCAGGAGACGTTTAAGGGTTTGGAGGTTGGAATGAAGAGAATGGTGATTGCGGTGGGTGATGGTGAGGAGTTTTATATAACGGAGAAGATGACGGAGGAGAACGTGACGGGGAGATAACGACGTTAACGGTGGAAGTGAA

**GAPDH**

**>KR361321**

AAGCAGTGGTATCAACGCAGAGTACATGGGGGTCATCGTTCTCAGTTTCTCGTCTCCTCTCTTGCTTTCAAAACTCTCAGATCGTCATGGGAAAGATCAAGATCGGTATCAATGGATTCGGAAGGATCGGGCGTTTGGTGGCGAGAGTAGCGCTCCAGAGTGATGATATGGAGCTTGTCGCCGTCAACGACCCCTTCATCACCACCGATTACATGACCTATATGTTCAAGTATGATAGTGTTCATGGCCAATGGAAGAATCATGAAATCAAAGTTAAGGACTCGAAGACCCTTTTATTTGATGAGAAGGCAGTCACAGTTTTTGGTATTAGAAACCCTGAGGAGATCCCATGGGGTGAGGCTGGTGCTGATTATGTTGTTGAGTCAACTGGTGTATTCACTGACAAGGACAAGGCTGCAGCTCATTTGAAGGGTGGCGCCAAGAAGGTCATCATCTCTGCTCCTAGCAAGGATGCTCCGATGTTTGTGATTGGTGTCAATGAGCATGAGTACAAATCAGATATTGACATTGTTTCGAATGCTAGCTGCACCACCAACTGCCTTGCTCCTCTTGCAAAGGTTATCCATGACAACTTTGGCATTGTTGAGGGTTTGATGACCACAGTGCACTCAATTACTGCCACACAAAAGACTGTTGATGGTCCATCTGCCAGGGACTGGAGAGGTGGAAGAGCTGCCAGTTTTAACATTATCCCCAGCAGCACTGGAGCCGCTAAGGCTGTTGGTAAAGTTCTGCCTGCCTTGAATGGAAAACTCACTGGGATGGCTTTCCGTGTTCCAACTGTTGATGTCTCTGTTGTGGATCTGACTGCCAGACTCGAGAAGGCAGCAACTTACGATGAGGTCAAGGCTGTAATCAAGCGCGAGTCTGAGGGCAAGTTGAAGGGTATCTTGGGTTATACTGAAGATGACTTGGTCTCAACAGACTTTGTGGGTGACAGCAGGTCTAGCATCTTTGATGCCAAGGCTGGAATTGCTCTGAATGAGAATTTCATCAAGTTTGTTGCTTGGTATGACAATGAGTGGGGATACAGCAACCGTGTTGTTGACTTGATCCGCCACATGTACAAGAGCCAGTAAGATGTTTATCCCTCAAATCTAGAGCCAGTCTCCATCAATGTGGTACATGGTCTGTTTGTCCTTAGTAGTTTGTGTATTTCTGATGGGAATAAAAGTCCGATACTTTGGTATTTGGGTCTATTGCTTTTCTGAATATGTTGTGTTGGTCTGAGGGGTACCCTACCCTCATTTTGTTGAGTTATGCTCTGAATCGGCTGAAATTTGAACTGTAACTTGTGATAAAATTGGCTGTGTTTGCAAAAAAAAAAAAAAAAAAAAAAAAA

**ACT**

**>KR361320**

ATGGGTCTCCATCAGAGAGCCCCTTCCGCATCTCCGCAACAACCGCCTTCTCTGATCACCTTCTCCGACGAGTGTTGTAGAAAATGGCTGACACTGAGGATATTCAACCACTTGTCTGTGACAATGGAACTGGAATGGTTAAGGCTGGTTTTGCTGGTGATGATGCTCCTAGGGCTGTATTCCCTAGCATTGTTGGTCGACCTCGCCACACTGGTGTCATGGTTGGGATGGGACAGAAAGATGCTTATGTGGGTGATGAGGCCCAGTCCAAAAGAGGTATTCTCACTTTGAAGTACCCAATTGAACATGGGATTGTCAGCAACTGGGATGACATGGAAAAGATCTGGCATCACACATTTTACAATGAGCTCCGTGTTGCGCCTGAGGAACACCCTGTGCTTCTCACTGAAGCCCCTCTTAACCCCAAGGCAAATAGAGAGAAAATGACTCAAATCATGTTTGAGACTTTCAATGTGCCTGCAATGTATGTTGCTATCCAGGCTGTTCTATCCCTCTACGCCAGTGGTCGTACAACAGGTATTGTGCTCGACTCTGGTGACGGTGTCAGTCATACCGTTCCAATTTACGAAGGCTACGCCCTCCCCCATGCCATACTTCGCTTGGATCTTGCTGGCCGTGATCTCACAGATGCCTTAATGAAGATTCTCACTGAGAGAGGCTATTCATTTACTACAACAGCTGAGCGGGAAATTGTGAGAGACATCAAAGAGAAGCTGGCATATATCGCTCTTGATTACGAACAAGAGCTCGAGACAGCCAAGAGCAGCTCTTCTGTGGAGAAGAGTTATGAACTTCCTGACGGGCAGGTAATTACTATTGGTGCCGAGAGATTCAGGTGCCCTGAGGTTCTCTTCCAACCCTCATTGATCGGCATGGAAGCTGCTGGAATCCATGAGACAACATACAACTCTATCATGAAGTGTGATGTGGATATCAGAAAAGACCTCTATGGTAACATTGTGCTCAGTGGTGGTTCAACTATGTTCCCCGGCATTGCTGATAGAATGAGCAAGGAAATCACTGCACTCGCTCCAAGCAGCATGAAGATCAAGGTTGTTGCTCCACCTGAACGGAAATACAGTGTCTGGATTGGTGGTTCCATCCTTGCATCCCTCAGCACTTTCCAACAGATGTGGATTTCGAAAGGTGAATACGACGAGTCTGGTCCTTCTATTGTTCATCGGAAATGCTTCTGAGGTTTTCCAGCTTGAAGTTATGTCTATCTATATGGTCTACAAGTGATTTGATGCATGTTTGTTTAGGCCAAATTGTGATGTCTCTACTTAATTGTTTTCAGGGATATCACAAACACTTTCCTCTTCTATTATTATGTTCTTCTCTATGTTTTATGTTTTCTGAGGCTGATTGGTGGTGTGAATTTGTCTGTGTTTTTATTTTATATCAGTGTTCTGGGCTTGTATCAGATATATTATGTAAACACTTCAAACATTTAGACTTGTTAGTAGCTGATGGTTTGCTGTGTTAAAAAAAAAAAAAAAAAAAAAAAAAAAAA

**APT**

**>comp78359_c0**

GTTTTTTATTTTTATTTGTTATATTTTATTTAAATAATGGTTTTTCTTAACTAAGTAAATGTACATAGGAGAATAAGCATGACATTCTCTTGACTATATTTTGTATCTTATTGGTGTCTGAGTTTCAGTATTTGGTCCCATGGGCTGGTCATCTTTTTCTTTCTGTTATGTCTATAAAATTCGTTTTGCTAATAAGTAAATTGACCAAGCTAAATTTTTGGTGACTTCTGTTGTGTGTGAGTCCTTATTTTGGGTGTGATAGAATATTAGTTTCCATAATTTTGTCTTAGGTATTGAAGCCCGAGGCTTTATGTTTGGTTCTCCACTTGCCATTGCCATCGGAGCAAAGTTCATTCCACTGCACAAACCTGGAAAGCTACCAGGCAAAACAATTTCAGAAGCCTATGCGCTTGAGTATGGTACTGACTGTTTGGAGATGCATGTTGATGCTGTTCAACCTGGTGAGAATGTGTTAATCAT

CGATGATCTGGTTGCAACTGGTGGGACACTTCGTGCTGCAGTAAGAGTACTAGAACGTGCTGGTGCAGAGGTTGTTGAATGTGGATGCATCGTTGGGTTGCCTAAGTTCAAGGCTGGCTACAGGATTAATGGAAAGCCAGTATATATGCTTGTGGAAGCCATTAGTAAAACATCATAAGTAGTGCCTCAAACATGTTTCCAGGAAAAGAGTAATGTTGTTTTCCGGGGTGAAGTCTGCAAAGCCAATGTAGCAAAGAAAGCTTTCATTTCATTGCTGAGGCAAGTAGATGTATCAATTACATCATCAGAAAAACTATAAATGTACCAAGTAGCTTTGCTTTGTAAGAGACTGAATGAATCACTGTGATAACATAACATTTTGGAGATGCATGTCTCTCTGATTGCATATATTTTATTCTTCCTTGATCTATACTCTGCGAAAA

**MDH**

**>comp84254_c0**

TCAGGTTTTGGATTTCACTGGACCGGCAGAACTGGCCAACTGTTTGAAAGGGGTTGATGTAGTTGTCATCCCTGCAGGGGTTCCTAGGAAGCCAGGCATGACTCGTGATGACTTGTTTAACATAAATGCCAACATTGTCAAGACACTCATTGAAGCTGTTGCCGATAATTGTCCAGATGCCCTCATCCACATTATCAGCAACCCTGTCAATTCCACTGTTCCTATAGCCGCAGAGGTTCTCAAGCAGAAAGGTGTCTACAATCCAAAGAAACTGTTCGGTGTTACTACATTAGATGTTGTCAGAGCTAACACATTTGTGGCACAAAAGAAGGGCCTTAAACTCATTGATGTGGATGTCCCTGTTGTAGGGGGTCATGCTGGGATTACTATTTTGCCATTGTTATCAAAGACTAGGCCTTCCACAACCTTCACTGACAAAGAAGTAGAAGAGCTGACTGCAAGGATTCAGAATGCTGGGACAGAGGTGGTTGAGGCTAAAGCAGGTGCAGGATCTGCGACTCTGTCAATGGCATATGCAGCAGCTAGATTTGCTGAGTCATCTCTCCGAGCCCTGGATGGAGATGCAGATGTATATGAATGCTCTTTCATTCAGTCTGATCTGAACGAGCTGCCATTCTTCGCTTCGAGAGTTAAGCTGGGGAAGGAAGGTGTTGAAGCTGTGATATCAGCTGATCTTCAGGGGCTGACAGAGTTCGAGGAGAAAGCACTAGAGGCCCTCAAGCCAGAGTTGATGGCCAGTATTGAGAAGGGTGTGGCTTTTGTGCAGAAGCAGCCCGCAGCATCAGGTTCCGCTTGATGATGTTTGTATATTTCTAACGGTCATGCGCGATACAAAAAGGGAATAACGACAGAGTGGATGCTCATGTCCTTTCAATTTTGCAGCTCATTAGGTTTTCTTGTTTCTACTTCCTTTGCAGTGGCTTTGTAAGACCATATAATTCCAGTTTTTGTTTATGTGATTGTATATCAATCATGTGACAAATTTTGGTTATGTAAGTGTACTGCCAAAGAAAAATGTGAGGCTTGGTATGTGATGCCTGTTGTGATATTTCAATTGTGAGACTTTTTTTTCACCTTACCATTTTTTTTTACCAAATACAGATGCTGTGGATTTGACAGAGTTTAACTTCTGAGTTTCTATGATGAAATTGGTATCTTCAATGTCAAGTTATCAACATTCAGGGAGCTTCTTGAAGTTCAAGACATCAAATAAAGTACGTAATTCTGATAACATTCAGTAGGCATGAAACCAGGACTTAGAAGGATGGGAATTGGACTTGGGAGGAGCACTTGCTAGATGGTAACACAGGGCATTCAATGACATTGGATCCTGAAGTATCCACACATAGATAAACTTGGTACAGTTGATTGTTCCCAGCTTCATCAGTGTTGCATTCTACTCCAGGTTAAGTGCGGCAAGGAAGTAGTCATGCTGATCGAGAACCGATTCCGAGCATGTTCCATGCTTCTCCCATTCATGTGCCCAGAAGGTGTTGCCATTGCTGCTGGGGCAGGCAAGCGATGGCCAGTTTGCTTGCATCCTACTATTCAAGTCGGCAATCTGCACATCATCGGTATGTAACCATATTACATTGACATGATTATGATCTAATTAGGGATGATTAAACAAAGACAAGCATGACAACACCTTAGATGGATTAAAAGGAGAGTTTTGATCACAGTTTGAAGGGTAAGATCCATCATTGAAGTTAGGCCAGAGGCCATGAATCCCAAAGTTTGCTGCTGGTTTCCCTGTGGATGGATAGCAACATGATTGCTGAATGTCACAGTAGGACCCTGGCCACTGCAATAACAAATATTGCCTGTTACTAATTCGATCAGTTATCGAAATATGTCGAATAGGTGTAAATACCAAATGCTCACCTGTTGAACAAAGTAGAAGAAATCAAAATTTTGGGAAGCTGATAAAAATGTGCATGAGAAAAATGCAAGAACATAGAGAAGGGAGTTGATGATAGTTTTCATGGTTGCTTTGATTTATCTACAATGCAAGAACTATATTTATATATAAAATGAAGGTGGAATGTTTTGTTTGGCATATTTGGCTTTGCCTTAAACAAAGTCTAGGATGAATTGCTTGATCATCTGACTATTTGTTTGTCCTGAGACTGAGTTGCTGTCTTCTTATTATTTA

**PP2A**

**>comp74046_c0**

TCAAGGCCGTCTCTTGGAAACAAGATCAAAACCAAAAACTCATCCAAATCGAGATCATTCCCAATTGCTTCTCGGAAACCCTAGCCGGTCGGAGATCGGGCCGGCGACGAGCGAACGATGCCGGCGTCGCATGGGGATCTCGACCGGCAGATCGAGCGGCTGCGGGAATGCAAATTCCTCCCCGAAGCGGAGGTGAAGGCGCTTTGCGAGCAGGCGCGGGCGATCCTTGTGGAGGAGTGGAACGTCCAGCCCGTTCGCTGCCCTGTCACTGTATGCGGCGATATCCATGGCCAGTTCCATGACCTTATCGAGCTCTTCCGCATCGGCGGTGATGCCCCGGATACCAATTACCTCTTCATGGGCGACTATGTTGATCGCGGTTATTATTCAGTGGAAACTGTGACTCTTTTGGTTGCCCTTAAAGTTCGTTATAGAGACAGAATCACAATTCTCAGAGGAAATCATGAAAGCAGGCAAATAACTCAAGTGTATGGTTTCTATGATGAGTGCTTGAGAAAATATGGTAATGCCAATGTCTGGAAGTATTTCACAGACCTCTTTGATTACTTACCCCTTACAGCGCTTATCGAGAGTCAGATATTTTGCTTGCATGGCGGTCTCTCCCCATCCTTGGATACATTAGATAATATTCGTGCTCTTGACCGCATACAGGAGGTCCCTCATGAAGGACCCATGTGTGATCTTCTATGGTCTGATCCGGATGACCGTTGTGGTTGGGGAATATCTCCAAGGGGAGCCGGCTACACATTTGGACAAGATATAGCTCAGCACTTTAATCACACTAATGGTCTCAGTCTTGTAGCTCGAGCCCACCAACTTGTGATGGAAGGATTTAACTGGTGTCAGGACAAGAATGTTGTAACTGTATTCAGCGCACCAAACTATTGTTACCGTTGTGGTAACATGGCTGCTATTATGGAGATCGGAGAGAATATGGATCAAAACTTCCTCCAGTTTGATCCTGCACCAAGGCAAATCGAGCCAGACACCACCAGGAAGACTCCTGATTATTTCTTATAATCGTCTCGTTTTTCAGTTCCGTTTGATTCTGTAGATTTGTGTTCGATGGAAATATGGTGGTTGTGTCACCGGGGGGATCTCGTCGTTCTTCTTCTGCCTGGAGGTTTTTGGATATTGACGAAGATCTGTTTTCGTATTCTATTCTTCTTTATGTGGATTCTCAGAGGCTTCTGTTGTTCTTCTTTTCCTTTGTATTGTAAAAGAAATTTAGAAAGCAGCAATGACAATTGATAATATTTCTCCTAATTTTCTTCCAATGTTTTCTGGCAGGTCTTCTAAATAATTTTGTTGGAGTTGTTCAAATGTGTTGGATGTTTTACAATCTCTTGTTGAATGGAAAAAGTTTTT

**TIP41**

**>comp79289_c0**

AAAAATAAAAGACAAAATAAAAACACAATTCATATATATATATATACCCTAAGCACTTAATTAGCTCAAACCATATGAAATAATGGACCAGTGTTCAGACACAACAAATCATCACTCTGATCAAACCAAGATATGTTGCATAAACAGCTAATTCATCAACAGAATGATATTAACATGTGTTCCACTTAAATCAGCATTTGATGTTTAAACATTTATCCATGAAGTTCTCCAGTTTCATCAATTCCCATCAAGACATAGCATAACACAGGATTACCGGGAAACACGAAGCTTTTGGATTTTATGAGCGACGACGGGAAGCGTTTGACTGATCAGATTAGGATCACCATATGCAGCAGAATCAGACGGAAAACCTTTGGCGGTTAAGCTTTGAAGTGTTGCTTCCCTCCAGCAGCTCTCACGTAGAATGATAGGCATCACATTTTCATCGGTAACAAAAGTACAATGCATTCTAGTATCCCTCAACCTCATAAGCACTCCATCAACCCTAAGCCAAAAACGAAGCAGAAGAAACCAACAAGTCGGCATCACTCTCACTTTGACAGTTAAGAGTGATACTCCATTATCAGCTAATTCATCTTCATACAAAACCACCTCATCATAGAAGAGTATTGGCTCTCTAGATGATAGTGCAGCAAGATCAATTTGTTGATCACAATCCTCCCAATGCAAGCTACAACAACCATCTTCCACACTTTCCTTTGGTGAATTTGGCACAAATATCTCACTGCCACAATATGGTGTTGTGAATGTATAGTCATAATCAAGAATCACTTGCTGAGAAGGTTTGCTCCTGAATTTCCACTTGGCTGCAGCAGGAACCTCAACCGGTGGCAATGCCTCCTGTTTCCATCCCATCAGAGCATCAAATGCATTGAAATGTATTTTAATGCCACTTTCAACATGCAAGAGAAACAAAGAACTGTCTCCAAAGACCATCTCTGGCAAATGTGAGGTCCCAAGCCTTTGCTCCCACTGATGAACCGCAGAAGAGCTGAGGATAGCGCGCTTAGAAGACTCGATGTGCCAGCCCTTGAGCCGAATCCCGCAGAGGCCATCGGGGAGAAGCTCCGCCCCGGCGGCCTTCAGCTCGCTCTCCTCTTGTTTCCATTCTTCTTCGCCGCCGCCATGGTCACCCGCCATTGCGCTCCGCTGGATCACGTTAAGCGTTTCGAAAACCCCAGCACTTCAATCTCTAACTACTCTTTTGAC

**GUSB**

**>comp87528_c0**

GGAGGGTGCAGTTTATATCAATCATAGCCAATAGCCATCCTTGGTGCCCTCCATCCTGTCATTTCATCGCACCATCTCTGGTTCTTATCTTTTGAAACGGTGAATCCAGATGAGAGTTTGACTTGAACTTACCAGTGTGGAGGGTGTAACAGATAGGGAATGAAGCTCCTCTAATCTTCTCCTCCTCTATCTCATCCCCTTCCCCTCTCTCTTTCTTGATCTCTTCCAAATCAAGGCTTAGGTCAAGGCATTAGCTCAACAATCTCCCCCGCGGGGAGACTAGTGCGAACGGAGGATTTGTTCTCTGGCTAGAATGTCGTTTGTTTCTTCTGTTCAGCTTCCTTTTTCTTCAAACTCTGGTTATAAGTTTTGGGAGGATCCGTCTTTCATCAAGTGGAGGAAACGTGATGCTCATGTGCCTTTGCAGTCGCATGATTCGGTTGAAGGATGTCTTAGATATTGGTATGAGCGCAACAAAGTAGATCATGTTATTTCTAGTTCAGCTGTGTGGAATGATGATGCTGTTTTGGGTGCTCTTGAGAGTGCTGCATCATTGGTTAAGGAGTTGCATTTTGTGAGGTCGCTAAGTGGCCAGTGGAAATTCCTTTTGGCCTCTTCTCCTGCAAATGTTCCTATCGACTTCTATAGTAACAGTTTTGATGATTCAGTATGGGAAACATTGCCAGTTCCTTCAAATTGGCAGACGCATGGTTTTGATCGTCCGATTTATACAAATGTTGTGTATCCCTTTCCTCTTAAGCCGCCACATGTCCCCTCCGACAATCCAACAGGCTGTTATAGGACATATTTTCATATCCCAAATGACTGGGAAGGTCGCCGAATCTTGCTTCATTTTGAGGCTGTTGATTCTGCATTTCTTGCTTGGGTGAATGGGGTTCTTGTCGGTTATAGCCAGGATAGTAGGCTTCCTGCTGAGTTTGAAATCACAGATTGTTGTCATCCATGTGGTTCAGATAAGGAAAATATTCTTGCCGTTCAAGTCTTCAGATGGAGTGACGGCTCATATTTAGAAGATCAAGACCATTGGTGGTTGTCTGGTATTCATCGAGACGTTCTTCTTCTTGCAAAACAAAAGGTCTTTATCACGGACTATTTTTTTAAATCAAGCTTCGCTGAAAATTTTCTTGTTGCAGAACTTGAGGTTGAAGTAAAGTTTGATATGATCCATGTGGATTCTGAAGATGTTGACATTTCTAGGTTTGCCATTGAAGCAGCATTATATGACAATGCAGAGTTGTCTGGCTACAGAAAGAATGAAGGTGTTGCTGATTTTAGTTCTTATAGCCCAGTGCTTCTAAAGCCAAAGTCACTGTGGAGTGGTTTTGGTTTTCCTGGTTATCACCTTGTTGGGAAACTAGAAATGCCAAAGCTATGGTCATGTGAAAATCCAAACTTGTACACTCTGGTTCTCACCCTCAAAGATGAATCTGGAAAACTACTAGACTGTGAATCATGCCAGGTAGGTATGAGGGAAATATCTCGGTCTCATAAGCAGATACTTGTTAATGGACAGCCAGTGGTCATTCGAGGTGTCAATAGACATGAACATCATCCATGTACAGGCAAGACAAACTTGGAAGCCTGCATGATTAAGGATTTGGTTTTAATGAAACAAAATAATATCAACGCTGTGAGGAATAGCCATTATCCTCAACACCCTAGGTGGTACGAGTTGTGTGATCTTTTTGGAGTTTATATGATTGATGAAGCCAATATAGAGACACATGGGTTTTCTCTTTCTTCTCAATTTAAGCATCCAACATCAGAACCAAGCTGGGCAGCCTCTATGCTGGATCGTGTTATTGGCATGGTGGAGAGGGACAAAAACCATGCCTGCATTATTTCGTGGTCATTGGGAAATGAATCTGACTATGGACCTAACCATTCTGCTCTTGCTGGTTGGATTCGAGGGAAGGACCCTTCTAGGCTTTTGCATTATGAAGGGGGTGGATCTAAAACATCATCAACCGATATCGTATGCCCCATGTATATGCGTGTGTGGGACATGGTAAAAATTGCTAATGATCCAAGTGAAACTAGACCTCTGATTCTGTGCGAGTACTCACATGCAATGGGGAATAGTAGTGGAAATATTCATGAATATTGGAAAGCTATTGATAACACATTTGGATTGCAAGGGGGCTTTATTTGGGAGTGGGTTGATCAGGCTTTATTGAAGGAGGGTACAGATGGATATAAATACTGGGCCTATGGTGGTCACTTTGGAGATACGCCAAATGACTCGAACTTTTGTTTAAATGGCCTTACCTGGCCAGATCGAACTCCTCATCCTGCCTTGCAAGAAGTTAAGTATGTCTATCAACCAATAAAGACTACTTTAATGGATAGCAGGATGAAGATAACAAATGCATTATTTTTTGAGTCAACACAAGGATTGGAGTTTAGCTGGCATCTTATGGGAGATGGATGCAACCTGGGATCTGGGGTGCTCAATGTTCCAGTAATAGAACCACAAAGTAGTTATGATATTGAGTTGGATTCATGTCCCTGGATTTCTCTTTGGGCATCATCTTCTGCAATAGAAATATTTGTAACAATAATTGCAAAGATGAAGCATTCAACTCGTTGGGTGAAGGATGGCCATATAATTGCTTCTTCTCAGCTGTGCTTACCTTCCAAAAGAAATCCTGGACCTCATGTTATTAAAATAGTAGACTGCGGTGGTTTAACTTCTGAGAATATCTCTGGCATTCTCACTATTAACAAGGAGAACAACTGGCAAATCAAGGTCAACAATAATACAGGAACTATCGAGAGTTGGGAGGTTGAAGGACATTTGTTGACTTGCAAAGGTATACTTCCCTGCTTTTGGCGAGCTCCTACTGATAATGATAAAGGTGGGGAGTCAAATAGTTATGCAAGTAAATGGCGGGCATGCCATCTTGATAAGATGTCAGTTCATACTACTCATTGCTCCA

TTGAACAACAGACAGATCATGTTGTGCAAGTGAAAACTGTTTATTTCATTGTTACTGAGGATCAAGATTTCCTGTCTAAAGGTAAAGACACCATTGATGAAACAGAGACAAGGAGTACAGTCTTCTTCAGGGTCGAGGTGTGCTACTGGTTCTATGATTCTGGCGATGTGATTGCTGAATACAATGTTAATCCTAACAGTGATCTCCCTCCTCTACCACGGATTGGGGTTGTGTTTCATGTCGAAGAATCCTTTGATAAGGTCACATGGTATGGGAAAGGCCCCTTTGAGTGCTATCCGGATCGGAAAGAAGCTGCCCATGTTGGCGTATATGAAAGCAGTGTAGCAGATATGCATGTGCCATACATTGTTCCTGGCGAATCTTCTGGCCGAGCGGATGTAAGATGGGTAGCATTTCAAGACAATGACGGATATGGCCTGTTCGCTTCTGTTTATGGTTCATCACCACCAATGCAGATGAGCGCAAGCTATTATAGTACTTCCGAGCTGGACAGGGCAACTCACAACAAAGACTTGGTCAAAGGAAATGACATTGAGGTGCATCTTGATCACAAGCATATGGGTTTGGGCGGAGATGACAGCTGGAGTCCGGCTGTCCATGATCAATACTTGGTGCCTCCAGTTCCCTACTCTTTCTCCATAAGATTGTGCCCGATTCGTCCTTCGCTTTCCTGTCAGGATATTTACATTTCTCAGCTGCCACCGTGATGATCATCCTTGTGT

**EF1-α**

**>comp77681_c0**

TACCAACATCCCAACCAGAAACACCATCTCATTAGATGATCACAAATATCTGCAACTCCAATAAAAAAAATCTACACCAAACCACTTTACACCTGACATACCACAACGCTAGAAAAATAGTATTAGTGTCCAATTCCATAGCGAACAAACTGGATTCAACCCAAAGATAAGAAAATCCATTCATAGTTTATAAATAATGACCAAACAAGGTACCGAGGGGCAACAGAAGTAAAACCCTCTGCAGCTCAAGTAGACTCAACCAGACAACCTGCGACAGAACATAGAGTTTTAAGAAACGCATTAGAAAAAACGAGGACCAATTGACAAAAGCTCCGCCACCGTCTATCTAGCACCCAGTTTTGCAACCAGATACCAGTCATCAATGGAGTTGACAAAACCCTAACAAATAAAGCATGGCAAACCAAAGTGCTTTCAACTACTAAATTTTTTTCGCCTTTTTTAGCTAAAAAGGCTGCAAAAGTGCTGCTCCGTCATTTCTTCTTTGCTGCGGATTTGGTAACCTTGGCTCCAGAAGGATCCTTCTTCTCAACATTCTTGATGACACCCACAGCAACAGTCTGGCGCATGTCCCTGACGGCAAAGCGGCCAAGAGGTGGGTACTCAGAGAAGGTCTCAACCA

CCATGGGCTTGGTCGGGATCATCTTAACAAGGCCGGCATCACCATTCTTCAAGAATTTGGGCTCCTTCTCGAGCTCCTTCCCAGATCGTCTGTCAATCTTGGTGAGGATTTCGGCAAATTTGACGGCAATGTGAGAAGTGTGGCAATCAAGCACTGGTGCATATCCGTTGCCGATCTGGCCAGGGTGGTTCATGATGATGACCTGGGAAGTGAAGTTAGCAGCCTCCTTAGCAGGATCCTCCTTGGAATTGGAAGCAACAAAACCACGCTTGAGATCCTTGACAGCAACATTCTTCACATTGAAACCAACATTGTCGCCGGGAAGGGCTTCTGGGAGGGATTCGTGATGCATCTCAACTGACTTGACTTCAGTGGATAGCCCGGTGGGACCAAAAGTAACCACCATACCTGGCTTGAGAACACCAGTCTCAACACGACCAACAGGAACAGTGCCAATGCCACCGATCTTGTAGACATCCTGGAGAGGAAGTCGAAGGGGCTTGTCTGAGGGCCGCTTTGGCTCCAGGATCATGTCAAGGGCCTCAAGGAGGGTAGGGCCCTTGTACCAGTCAAGGTTGGTGGACCTCTCAATCATGTTGTCACCCTCGAATCCAGAGATGGGGACGAAGGCGATTTTCTC

AGGGTTGTATCCCACCTTCTTGAGGTAAGAAGATACTTCCTTCACAATTTCATCATACCTGGCCTTGGAGTACTTGGGGGTTGTAGCATCCATCTTGTTACAGCAGCAAATCATCTGCTTCACACCAAGGGTGAAGGCAAGCAGGGCATGCTCACGAGTTTGTCCATCCTTAGAGATACCAGCTTCAAAACCACCAGTAGTAGAGTCAATGATAAGAACAGCACAGTCAGCCTGAGAGGTACCAGTAATCATGTTCTTAATGAAGTCACGATGGCCAGGAGCATCTATCACAGTGCAGTAATACTTGGTGGTCTCAAACTTCCACAGAGCAATGTCAATGGTAATACCACGCTCACGCTCGGCCTTCAGCTTGTCAAGCACCCAAGCATACTTGAATGACCTTTTGTTCATCTCAGCAGCTTCCTTCTCGAACCTCTCAATCACACGCTTGTCAATACCACCAAGCTTATAGATGAGATGACCAGTGGTGGTAGACTTCCCAGAGTCCACATGGCCAATAACCACAATGCTAATGTGAACCTTCTCCTTTCCCATGGCTAAACAAAGCTAAAAACGAGAACTAGAGATCAGATCGCCAGCGGCCGAAAGCTTTGCTCAGAGGTGAGAAACCCTTGAAGTGTGCTGGTG

**UBQ**

**>comp74534_c0**

CAATCAAAACCATGCTAAACAAACACTACTCCATAATAATAATAATTATGATCAATAAAAGAGAATAAAGAGAAAGTAATAGAGGCTAGTTATGCATTGTCTATAAACAGTTTGAACAATAAACAGTACACAGCTTAGTAAAAAAGGGTATTTACAAGGAGAAGGTGAATAATCCTGGCAAATCATACTCAGTATAAACGAGAGCAAAGATGGATTAAAAGTTGGTAGCTATTGACCAATATCTACTTATTCCATGCTATGAATAAGAGGATCAATCTCTGGGTCTTGTCATTTTAAAAGCTTAACGCAAACGCAAAGAAATGTTCGGTTTTACACACCCTCATAATAATGGGCTATATATAATGAAGAAAATCACATTGTCGAAAAACTAATGACCGGGATTCCCAAGGAGGCGCTCAACTGCAGCATGGACATTGCCGGCGGTAGCACTCAGGGCTCGGATGTTCTCCTGTGTGTCGAAGAAGCCCATTTCTTGAAGCTGAGATAGCTGCGTTGCATAAAGCTCTTCGGGAGGCACATTAGATGGATTTGGCATGCCAAGGCCACCGGCCCCGAGTCCACCAAACAAGTTCACCAACAAATCAAGCCCCATGTTGTTCGGTGCGCCAGTGCCATCCCCACCCTGGTTCTGTTGCTGATTTGGCTGTTGTCGGCCCAACTGTGATGAAAATAGTCGCTGGAAGGATAGAAGTTGCTGCATAGTCTCAGGAGAAGACAGCTGGCGAAGAAATTCCGGGTTTTGCAACATCTCTCTCAATTGTGGGTTAGCCTCCAACAAAGGACCCATTTGAGGTCCAAGCATCTGGTTCATAAATTGCGGGTTGGACATAAGGCTTTGCATCCACTGCATCATGGCCGG

ATTCTGCAGAATCTGATTCAAGGAAGATGGATCTGGCAAACCACCAAGCATTCTGTCCATGTCTTGAACATTATTGAAACCACCCAACCCAGCAATCCCAGGTGACCTTGTATCTCCAGATGTTGCCCTGGTATTCCCTGCCGGTGCTGCTCCAGTGGCATTGCTCCAAGGATTAGGAAGTGGATTAGCATTTGGTGCAGGAGATCCACCAGTTTGTTCCAAATTGGTGTTTGAAAGATTTGAGGATCCATCACGAGACTGAGCAGAACCCTGATTCCCCAAAAGAGCAGCAAATGGGTTTGCCCCTGCATCATTTCCTGTTTCCCCAGCCATTGTTGTCGCATTCAGAAAAGGTTCCTGAACATTTTCATACATGCGGCGAAGCATATTAAAGCCTTCTGGAGTAGCTTCAATGTTGCTCATAGCCCTGTCTGTGTTACGCATCATCTCTCTCATCAGTTCAGGATTCCTGGCTGCTTCAAGAGTTTGTCTAAGAATGCTAGGATCATTAAGGACATGACCAAGTTCAGGATTCCGGTCTATCACATCACGCATTTGAGGATTACTCATGATTAAATTTCGCATTAGATCAGGATTATTCATAAGATTTTGAATGGCTGGCATGTTCATCATTTCCCTCATCAAATTGGGATTTTGAGTCAGCTGTTGTTGAACCTGACTGAAGTCTGGCAATCCAGTTCCAAATGGACTAGATCCCCCACCACTCCCAAACAGCCCATTAAGACCAAGACCAGGGAACTGTGCACCTCCAGAACCAGCTCCCCCATATCCAACACCTTCATTGGAACCAACCCCAACGTTTGGAGTCCTAGAAGCTCCAAGGTTGGCAGCTGGTGTATTTGATGATGCAGATGATGCTGGAGCGCCACGGACCAAATGAATAGTGTGATCAGACTCCACGCCATAGCTTTGAAGGGTTTGCTCATCCTTCAAGACGCGACCTTTATAGATCAGCCGCTGCTGCTCAGCCGGCACATCGCAATTCCCAGCAACGACGACCTTGAAAGCCCCGACGGTGACATCGAGGGGAGTCTGGAGAGAGAACTTAGAACCATTGGAGCAACGGATGTGGACGGTAACCGAGCAGCCGCCTTCAACGCCGCTCGAATCGCCGTCGGCACCCATAGGAGATCGGAGAACACGTAAAAAAAGCCCTAGAGAAACCAAATCGGAACGGATCTGATGGCTAGGGTTTGGATTGGCAGAGCTTTGGCCTCGAAGAAGGGAAGAGGCGAGAGATTTCTTCGAAGAGCGGATAGGTAGCAAAGGATGCCTTCGCACTTCCCAAACAAGGAAAAAATAAACAAGAGATTTTATTTATTGCTTTCCTGCAATTTCTCTGACTTTTTATCAAAAACAACCCCTGAC

**PE2.1**

**>comp86916_c0**

CCGGAAAAACATGTGCAAGCCCAAGGAAAATTAAAGAAAGCCAAAACTTAGGCAAAGGGGCCATCACCAACCCCATCACTCCCCCCAAAGCCACTGTCATCCTAATTTCATATAAACACTCCATTATTTTATTATCAAATCCACCATCAACGAGAATCATCATCTCCAAAAAAATTTCATCTATAAGTAAGTCACAAACCAAGCAACCACAACCACAAACACAGAAACTCAAGCTTTCATGGCACCAACCAACCCATGCAGCCATCTTCTAGTCACAGTGCTCTCCTTACTAACCATCATCCCCATCTCAACATCTTTTCCCACCACTCAAAGTGATGATCCCTTGCTTAAAATCCTTCACACATCCTTAACCCAGCTCAGCACCACTATCAGCACTGCCACAAACTTACACCGCCGCATCAACGAGCAAAAACAGCAGGCAGAGCTCGAGGATTGCTTGCAACTCCTAGACCTCTCTCGTGACCGTGTGCTCAGCTCGGTCCGAGCTATCACTAAAAGCGCGCACACAGATGCGCGCACATGGCTCAGTGCTGTTCTGACTAATTATGACACTTGCCTTGATGGGCTAAATGGTTACGTGAAACTCTCCATGGAATCTCAACTCAAGTCCTTGATGAAATCCACGAGCGTGTCACTGGCCTTGCTGGCGACCAGTGACGACGACGACGATGTTCTCACTCAGGTGGTCGAGTTCCCGTCCTGGATATCTAAGCGTGATCGAAAGCTACTCGAAGCTCGAACGCCGAAGGCTGTGCAAGCCAATGTTGTTGTGGCCAAGGACGGGAGTGGGAAGTTCAAGACGGTGCAAGAGGCATTGAATTCTGTCCCTGATAATGGCAATACAAGGTATGTTATTTATGTGAAGAAAGGTGTTTATAAAGAGACTGTTAGGCTTGAGAAGAAGAAGAAGAACGTCATGATCGTTGGTGATGGTATGGACGCCACTGTCATCACCGGTAGTCTCAACTTCGTTGACGGAACCACCACCTTCAACAGTGCCACGTTAGCTGCGGTTGGTGATGGACTGATTCTCCAGGATCTAAAGATAGAGAACACTGCCGGAGCGATAAAGCACCAGGCGGTGGCTCTCCGGGTCGGAGCCGACCGATCAGTGATCAACAGGTGCAACATCGAGGGCTATCAAGACACGCTTTACACGCACTCACTGCGTCAATTCTACCGTGACAGCATTATATCCGGTACAGTGGACTTCATCTTTGGTGACGCCGCTGTTGTTTTCCAGAACTGCAAACTCGTGGCACGGCGTCCCATGGACAACCAGCAAAACCTTGTGACAGCTCAAGGGAGGATAGACCCCAACCAGAACACTGGTACTTCCATACAGAACTGCCAAATAGTTCCCAGTTCTGACTTAAAGCTTGCAGCAGGGGCTATAAAATCATATCTTGGTCGGCCATGGAAGGAGTATTCTAGGACTGTGTTCATGCAGTCATATATAGATTCACATATTGATCCTAAAGGGTGGTTGGAGTGGGATGGACAATTTGCACTAAAGACTCTGTTTTATGGGGAGTATATGAATAAGGGGCCTGGGGCTGGTACTGGTGGCAGGGTTAATTGGCCTGGTTATCATGTGATTACTGATGCTAATATTGCTAAGTCATTTACTGTGGCTAGTCTTATTCAAGGAGGAACTTGGCTCAAGGACACTGGAGTAGCTTTTACTGAAGGACTTTGATCTCTTGATGCAGTCGCTTGTAACTTTGTAATGTTCCACTGGAGAGTGATCCTTTAATTTTGTACTTGAATTTCTGGTTTGTGGAAAATAAGAGGCCGGTGCTTTCTTTTCACTTCTTTTTATTCCCCTGTTTTTACTGGATAGGCATATTGCTTTGTAGTTTAGCATGTTTAAGTTCCCTGTTTTAGTTTTCTTCACATTGAAGTTAGGTTGATTATACGGTGTCAGCTTCTCAAAAAAGGAGATTTGATAAGTGTGAGAACATTAAAGGAGATAGATAAGTCAAACTTATCAAAGGTGTGGACCTCGCCCATTACTAAGCTTGATTAGAATACTAAAATGAATTTGATTTCCTCCCTTAATCTCTCTTTTTCTTGTCAGGTTGCTCTCAAAACATAGTTGTAATGAGGAAGAGAAGAAATTCCGGTACTATCACTTTATGTTTGTGGACCCCACTCTTTAATTGGATCGGGCCGATGTAATTTTTTTTAATTTTAACCATTGATTTTAAATTATTTATAACTCTTGTTCTTATCTCTAGCTAGTGAGTAATAGATTTGTTTTAAAATGTTTTATAGATTAAAAGTGTGGTAGACTGGTGTGGATTTTGAAAGGCCGCCAATAAGTGTTTTTTTTTGAAAAAATAGATAAACCACATGTATTAAAAAAAGGAGGGCTGCCAATAAGTATTGTTTTGTACTGTTTGAGTTTTTGTCATTGTCTGTTTTTGTATAGCTGTATTTTATATTTTGAACTTTTTATATTCTTATACTGTATATATACTCTGTAAAGTCTATAACCTTGTGACTGATCATTTGGTTTGTTAGAATTTATCTTCTCTAAATCAGATTATGAGGCTTGTATGGGTTCGCATGGGTCTGCGGTCCATTTGTCCAGCGTCGAGTCCGGTCAGGCCAGGGTCTGGTGCGTGACAAAAGGGAAGAATAATAATGATGCTTTCTTCCATATAATCAATCAAGGTTTTCTTCGAAAGATCATTTTAACATTGGAGATCAAGATGCTATAACTTTGTACATGTGACTTGCTTTTTTAAAATATTTATGTTGGTTATATAGGTTTGTTTGCTTGTTTTTTCTCTCAATTATTTTATTATACAGTATATTTGAGAGAGCCTTAAGATTTTTCTTTCTGTAGGTTTTCCTGTAAATTAATCTTT

**PE53**

**>comp76893_c0**

TAGAAGAAAAATGACCAAAATCACTTGTTAATTTCAATGATCTGGGAATCGAATGTAACAATGTACAAGTGTGGAATTATTAAGTGAAGTCAAGCAACATTATTGATGTTAAAAATCAAGAATTAAGATGATCAAATCTTGATCCAATCAGAGCCGTCAATGAAGCCTAGTGATATGAATGGCTTAGCTTCTTCATCTGTGAGCTCTCTAGACCATGCCACTCTGCCTGCATAGCTTGCTCCTGGCCCATAACACTTATACTGTCCATAAAACACAGTCATTTCTCTGTTAGGATCTCCCCAGTTATACCATCCCTTAGGAATAATAATGTTATCCATGTAAGTGTAGGCAAAGATAACTCTGGAGAAAGTGCCCCAAGCTCTGCCTAAATACAGAGCACCAGATCCAGTCACTTTGCAGTTCACAAATGAGAATCCAGTGTCCTCCAAAATACTCATTCTGTTTTGTGCTGTCAATGCTCCATAGTTCTCTGCTATTGCATGAACATGACACCCCTCAAACAGAGATAAAGCATTGCCGAAGATAAAATCGACAGATCCTTCAATATAACAGTCTTTGTAATAATGTCTGCCAACATGATCATAGAGTGTGTCTTGAGCTCCCAAGAACTTACAACCCACAAAGGTTGCTGTGTCTCCAGATATCCTCAATGCCACTGCTTGTTTTCCTATTGCTCCTGGTGGTGGCACTGGTGTAGTGTTCTTAAATGTGATGTTCTTGGCTATGAAATAAGGTGAATTGATAGCAAATGTAGCAGAGTTGAATGTTCCAATGGGTTGTCCTTTAGGACCCAAAGTTTGAGCAGTGTCCCCCCATTGAACAACAGTAACATCAGCTCCATCTCCTTGAATGGTTATGAATGCTCTCATTGGTGAGATATTCACCTTTTCTGTATAAGTCCCTGCATTAACTTTGATGACAACTCTGACAAGATTAATGAAAGGAAGTGAATCAATGGCATCTTGAATGGTGGTGAAGTCCCCAAGTGAAGGGTTCTTGTCTACAGTGAGAGTGTAAGAAGGGAAGAGCTTGTTGAAGGGTTTACCAAACAGAGTGTGTCTTAAGCCACCCATGAACTTGATCCATTTAGTGAACTGAACTTCAATCAACTTAGCTCTAGTTTGGTTGGGTTGAAACTGGTCCCAAGGTGGCCGGACACCTGGCCTGAGCTTCTTTGTGTGGTTGCCGGAGACTTCACCGGAGATGAAAATAATGAAGAAGAAGAAGAGAATGATGGATGGTGACAGGGATTTGGAACTGGAATGAGACATTGTTGACACTGAGAAAGGTGGGATGTGTAGTTGGAGTTTTTTGAGGGGATTTTAATGAGTTCACGGGATGGAGAAGTTGGGGTCACAAGATGAATGAAGGTACGTAGATTAAGCACTCATGTTCTGGACCTCTTCGTCCAGAATACTCTCATCGATCTCTACGTGCAG
